# Placenta Prævia

**Published:** 1879-10

**Authors:** John Hauenstein


### CLINICAL REPORTS.

### PLACENTA PRÆVIA.

REPORTED BY JOHN HAUENSTEIN, M. D.

THERE are few cases that arouse the physician to the full comprehension of his responsibility to his patient, more than so serious an accident as a case of placenta prævia centralis, the peril of life to the woman being so great, that it has been pronounced by the most eminent authorities to exceed that of some of the most malignant fevers. A case of placenta prævia, therefore, occurring in the practice of a physician, always taxes, to the utmost of his capacity, all his skill, resources and presence of mind. For this reason, and the fact of the case being typical of central implantation, I ask, respectfully, to be allowed the necessary space in your valuable journal to lay it before your readers.

*Case:* I was called in haste on the evening of July 28th, to Mrs. H., mother of seven children, and now about completing her seventh month of pregnancy, having ceased to menstruate December 17th, 1878. I found her in bed, somewhat alarmed and excited in consequence of having had a sudden gush of blood, issuing from her vagina, for which she could not account. The first sensation being that of warmth and of a fluid, as though the waters had broken, when she immediately took to the chamber, and passed a quantity of blood, amounting to about twelve ounces, as near as I could judge upon inspection. Although but thirty minutes elapsed from the time she was taken with flowing until my arrival, the hemorrhage had already ceased. A digital examination gave me no information other than that the blood had issued from the vagina; the os uteri being almost beyond my reach, and not dilated more than the size of my finger. Rest for a few days was enjoined, and iced lemonade ordered. On August 19th I was again hastily called, and found a repetition of all the essential circumstances as on the first occasion; the hemorrhage being somewhat less than the first time. On the 22d of August she had a third hemorrhage, differing not materially from the last preceding.

The responsibility began to weigh upon me, and I informed the husband that preparations for the safety of his wife had now to be made. He unhesitatingly entrusted the management of the case to me, and like a tender husband enjoined me to do my whole duty.

I therefore called on Dr. White, whom Mr. H. had suggested as counselor, when the programme of our procedure in the case was discussed and determined.

Accordingly, on the evening of August 25th, at 8 o'clock, we met at the house of the patient, and proceeded to induce premature labor by the introduction of a No. 10 catheter between the uterus and the membranes. This was followed immediately by a copious flow of blood, so much so as to induce us to apply the tampon at once, which we had in readiness, and which was made of small bundles of absorbent cotton, tied, at intervals, to a ribbon, like a *kite tail.* A compress and the T bandage were applied to secure the tampon in place. Right here it may be in place to say that the woman is predisposed to faintings, and had already, during this stage of the proceedings, fainted several times.

Thus were the preliminaries of the battle commenced. The patient passed a quiet night, and beyond the withdrawal of the urine by the catheter, the physician in attendance was not disturbed from his night's rest.

At 7½ o'clock on the morning of the 26th, an examination was made, the patient having had several insignificant pains a short time before, but no signs of hemorrhage were discoverable, the patient being comfortable, with no signs of commencing labor. Dr. White had an urgent call to be absent from the city for a few hours.

At 9 o'clock the first pain, indicating that labor was beginning, took place, the effect of which was a sudden loss of blood, made evident by the expulsion of clots from under the compress and bandage, and by the saturated condition of the same. The latter incident may, however, have been in part due to the liquor amnii.

There being no certainty as to the time when active interference would become necessary, I sent for Dr. Rochester, with whom Dr. White had already an understanding about the case, and had expressed his wish, in case of his absence, to have him summoned. But the pains subsequently were inconsiderable, occurring about every fifteen minutes, but yet gave occasion to the oozing out of some blood.

About 12 o'clock noon, Drs. White, Rochester and myself being in attendance, it was decided to remove the tampon and, if admissible, to deliver the child. The tampon being removed, the os uteri was found favorable for the manipulation of turning and delivery of the child, which was accomplished in a short time with but very little loss of blood to the mother. As soon as the operation was begun, a teaspoonful of Wyeth's fluid ext. of ergot was given the patient, quickly followed by a hypodermic injection of the same, and again repeated in about fifteen minutes; also injections of brandy, hypodermically, were given several times. After delivery, to avoid the greater danger incident to a case of placenta prævia, which is *post partum* hemorrhage, a plug, or rather a tampon, of absorbent cotton, saturated with cider vinegar, was applied to the patulous os and secured, the uterus having firmly contracted. So far everything promised well; the mother was comparatively comfortable and of good cheer, the child was crying lustily, and we doctors congratulated ourselves on the successful termination of the case, and that we could not very well improve upon the method employed.

This promising condition of our patient continued for about three-quarters of an hour, during which time, however, napkin after napkin was removed, considerably saturated with blood; although not very large, this hemorrhage, after the lapse of about three-quarters of an hour, told upon our patient; her pulse became feeble, her extremities cold and clammy, and large drops of sweat pouring out upon her face. Being now left alone with the patient, I began to fear for her safety. Resort was had to stimulants, the foot-end of the bed was elevated, the vaginal plug was removed and another, saturated with a solution of *persulphate of iron*, substituted, which suppressed the hemorrhage completely. In the meantime I sent for Dr. White.

Several hours, however, elapsed. It was 4 o'clock in the afternoon before reaction took place, and our patient was out of immediate danger. After this, the patient made an excellent recovery. The only drawback for the first few days was a persistent vomiting, which, however, yielded to iced champagne. She sat up in an easy chair on the tenth day for half an hour.

*Remarks:* There are two points in a case of placenta prævia to which I desire to direct attention. The *first* is the inefficiency of the ordinary tampon to prevent hemorrhage on the accession of labor pains. The vagina, being a yielding cylinder, cannot be plugged with an unyielding tampon, successfully, against loss of blood from the uterus, except there be no expulsive efforts or pains. Because the force exerted upon the blood above the tampon in labor pains is such as to force a dilatation of the vagina beyond the tampon and the consequent escape of blood. I advocate, therefore, the use of the air-ball, or, what is yet better, the colpeurynter, to be introduced through the speculum, with a moderately thick layer of absorbent cotton drawn over it, and large enough to cover it after being inflated or distended with water. This tampon yields, and can be filled to the capacity of the vagina without its withdrawal. It can be withdrawn and replaced rapidly, which under certain circumstances may become necessary. The *second* is the post partum hemorrhage, dependent, in my opinion, upon the tardiness with which that portion of the uterus, upon which the placenta was implanted, contracts, and the numerous and large blood vessels with which it is supplied. To prevent such hemorrhage, the application of a plug, saturated with a solution of the persulphate of iron, *is the remedy*, always keeping in mind, however, that the contraction of the uterus, to prevent internal hemorrhage, be attended to.

